# Seasonal Variation of Overall and Cardiovascular Mortality: A Study in 19 Countries from Different Geographic Locations

**DOI:** 10.1371/journal.pone.0113500

**Published:** 2014-11-24

**Authors:** Helena Marti-Soler, Semira Gonseth, Cédric Gubelmann, Silvia Stringhini, Pascal Bovet, Pau-Chung Chen, Bogdan Wojtyniak, Fred Paccaud, Dai-Hua Tsai, Tomasz Zdrojewski, Pedro Marques-Vidal

**Affiliations:** 1 Institute of Social and Preventive Medicine, Lausanne University Hospital, Lausanne, Switzerland; 2 Department of Epidemiology and Biostatistics, University of California San Francisco, San Francisco, California, United States of America; 3 Institute of Occupational Medicine and Industrial Hygiene, National Taiwan University College of Public Health, Taipei, Taiwan; 4 National Institute of Public Health - National Institute of Hygiene, Warsaw, Poland; 5 Department of Hypertension and Diabetology, Medical University of Gdansk, Gdansk, Poland; 6 Department of Internal Medicine, Internal Medicine, Lausanne University Hospital, Lausanne, Switzerland; FIOCRUZ, Brazil

## Abstract

**Background:**

Cardiovascular diseases (CVD) mortality has been shown to follow a seasonal pattern. Several studies suggested several possible determinants of this pattern, including misclassification of causes of deaths. We aimed at assessing seasonality in overall, CVD, cancer and non-CVD/non-cancer mortality using data from 19 countries from different latitudes.

**Methods and Findings:**

Monthly mortality data were compiled from 19 countries, amounting to over 54 million deaths. We calculated ratios of the observed to the expected numbers of deaths in the absence of a seasonal pattern. Seasonal variation (peak to nadir difference) for overall and cause-specific (CVD, cancer or non-CVD/non-cancer) mortality was analyzed using the cosinor function model. Mortality from overall, CVD and non-CVD/non-cancer showed a consistent seasonal pattern. In both hemispheres, the number of deaths was higher than expected in winter. In countries close to the Equator the seasonal pattern was considerably lower for mortality from any cause. For CVD mortality, the peak to nadir differences ranged from 0.185 to 0.466 in the Northern Hemisphere, from 0.087 to 0.108 near the Equator, and from 0.219 to 0.409 in the Southern Hemisphere. For cancer mortality, the seasonal variation was nonexistent in most countries.

**Conclusions:**

In countries with seasonal variation, mortality from overall, CVD and non-CVD/non-cancer show a seasonal pattern with mortality being higher in winter than in summer. Conversely, cancer mortality shows no substantial seasonality.

## Introduction

Hippocrates already recognized that mortality is higher in cooler than in warmer months [1]. In Europe, seasonality of mortality accounts for 16% more deaths in winter than in summer, making it a public health issue [2]. Mortality from respiratory diseases exhibits a clear seasonal pattern that has been attributed to influenza and other infections [3]. The same seasonal pattern is also observed for cardiovascular disease (CVD) mortality [4]–[6], possibly due to seasonal changes in temperature[2], [3], in cardiovascular risk factor levels [7]–[9] or to exacerbation by other conditions such as influenza [10], [11]. The scarce work conducted so far on the seasonality of cancer mortality suggests no or little seasonal pattern for these diseases, consistent with long gaps between exposure to risk factors and outcomes [12].

Most studies on the seasonality of mortality by cause of death have focused on a single country [6], [13], [14]. In this study, we aimed to establish whether a seasonal pattern in overall and cause-specific (CVD, cancer and non-CVD/non-cancer) mortality exists at the worldwide level. We thus conducted an observational ecological study using national case-specific mortality for 19 countries from different geographic locations, including broadly different latitudes. Our hypothesis was that if a true seasonal pattern exists, then it should be mirrored between the Northern and the Southern hemispheres.

## Methods

### Data

Countries were selected based on 1) the availability of cause-specific mortality data at national or regional levels; 2) the availability of information on time of death; 3) the availability of an email address for the online source of data; and 4) the possibility to communicate in English, French, Spanish, Portuguese or German with the managers of the sources of the data. In addition to our research among the available web-based mortality databases, we took contact with several persons associated with death registration in several Latin American and African countries, but only very few responded.

Cause-specific mortality data from 2000 to 2010 were collected from national statistics agencies or their representatives in 19 countries (see **table S1** and **table S2**). Chile exhibited large variations in the reported numbers of deaths in three consecutive months (approximately 7000 deaths in January 2006, 3000 in February and 7000 in March). In order to be on the conservative side in the case these differences reflected some error, we excluded February data from our analysis. Cause-specific mortality data were not available for France and New Zealand and these two countries were included only for the analyses of overall mortality. Mortality from non-CVD/non-cancer was obtained by subtracting the sum of CVD and cancer deaths from the total number of deaths. This was done because for some countries a more detailed information regarding the cause of death was lacking (i.e. infectious or external). For each country, the observed numbers of deaths per month were calculated as the average of the monthly numbers of deaths over the whole study period. The expected numbers of deaths per month were obtained by dividing the average total numbers of deaths per year by 365.25, and by multiplying the results by the numbers of days in each month, considering 28.25 days for February. For all country-month pairs, the observed/expected mortality ratios were computed; the closer the values to one, the more concordant are the observed and expected values. A value greater or lower than one indicates more or less deaths than expected, respectively. Each country average latitude was obtained from the Central Intelligence Agency World Factbook [15] except for Scotland and England & Wales, where mean latitudes were computed using the southern- and northernmost points (+57 for Scotland and +53 for England & Wales). Northern and Southern latitudes were designed by a plus (+) and a minus (−) sign, respectively.

### Statistical analysis

Statistical analyses were performed using Stata 12.0 (StataCorp, College Station, Texas, USA) and R Development Core Team (2011) [16]. As seasons depend on geographical latitude, three geographical locations were defined: Northern Hemisphere, close to the Equator, and Southern Hemisphere (**table S1**).

Seasonality of mortality was assessed using weighted fixed effects models including a cosinor function [17]. First, country-specific models were performed. Then, specific models were applied according to a country's geographical location (Northern Hemisphere, close to the Equator or Southern Hemisphere). For each country location, a joint model including a single intercept between countries was applied, assuming that the expected mean country-specific ratio is 1, and including sine and cosine functions:
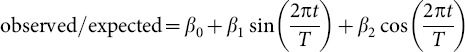
where observed/expected is the vector of ratios between observed and expected numbers of deaths per country-months, 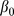 is the estimated mean ratios, 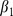 and 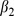 are the associated coefficients of sine and cosine functions, respectively, 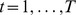 is the time period, corresponding to the monthly data, 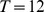 is the number of time periods described by the sinusoidal curve to assess yearly seasonality with one minimum value (nadir) and one maximum value (peak) per year. Details for the estimation of the amplitude, peak and nadir are shown in the **appendix**. The percentage of the between-month variance explained by the sinusoidal curve was estimated using the R^2^ measure. Likelihood ratio test was used to assess statistical significance of seasonal variations.

## Results

### Mortality data

**Figure 1:**
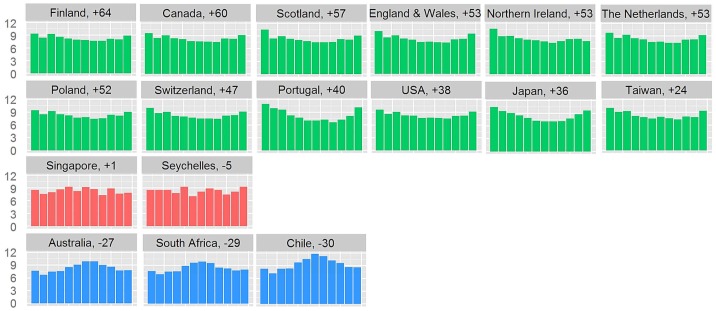
Distribution of the proportion of cardiovascular deaths (y-axis) per month (from January to December, x-axis) by country. The numbers close to the country names refer to the mean latitude of a country. The countries are positioned according to their latitude with the northernmost countries on the top. Countries from the Northern Hemisphere close to the Equator and Southern Hemisphere, respectively, in green, red and blue. No data available for France and New Zealand.

Overall, 54,547,005 deaths from 19 different countries within the period 2000 to 2010 were analyzed. Time intervals of 1 to 10 years per country were covered. The distribution of the proportion of CVD deaths per month by country is illustrated in **Figure 1**. Distributions of overall, cancer and non-CVD/non-cancer mortality are illustrated in **Figure S1, Figure S2 and Figure S3**.

### Seasonality

The estimates of seasonal variation (peak to nadir difference) and the percentage of variance explained by the model (R^2^) using cosinor regression for each country are summarized in **table 1** according to each broad cause of death. A seasonal pattern in overall, CVD and non-CVD/non-cancer mortality was found for most countries, with the exception of the Republic of Seychelles (no association) and Singapore (associations weak or null). The percentage of the between-month variability explained by the cosinor model was higher than 75% for overall and CVD mortality for most countries except for Northern Ireland, Republic of Seychelles and Singapore. For non-CVD/non-cancer mortality, the percentage of variability explained by the model was of a similar magnitude in the same countries. On the other hand, for cancer mortality, where a seasonal pattern was observed in only 7 countries and peak to nadir differences were not higher than 0.07, the percentage of explained variability was substantially lower, suggesting that the use of a model taking into account seasonal variation was not necessary.

**Table 1:** Total number of deaths, seasonal variation (peak-nadir) in the ratio of observed to expected deaths, and percentage of variance explained (R^2^) by the cosinor function for overall and cause-specific mortality.

	Overall		CVD		Cancer		non-CVD/non-cancer	
Country (N; mean latitude)	Peak-nadir	R^2^	Peak-nadir	R^2^	Peak-nadir	R^2^	Peak-nadir	R^2^
Finland (487,942; +64)	0.142***	0.924	0.185***	0.943	0.032**	0.562	0.165***	0.887
Canada (2,284,419; +60)	0.156***	0.960	0.216***	0.968	0.042***	0.916	0.199***	0.943
Scotland (618,292; +57)	0.220 **	0.784	0.243***	0.834	0.052	0.730	0.322***	0.728
England & Wales (5,652,495; +53)	0.248***	0.880	0.268***	0.931	0.043***	0.863	0.376***	0.838
Northern Ireland (160,000; +53)	0.200	0.593	0.229	0.614	0.031	0.102	0.302*	0.673
The Netherlands (1,511,810; +53)	0.175***	0.893	0.250***	0.974	0.027	0.562	0.231***	0.793
Poland (2,258,014; +52)	0.142***	0.941	0.215***	0.971	0.024	0.596	0.133***	0.859
Switzerland (487,635; +47)	0.198***	0.885	0.254***	0.920	0.033***	0.810	0.265***	0.829
France (5,947,305; +46)	0.189***	0.851	-	-	-	-	-	-
Portugal (735,340; +40)	0.334***	0.855	0.466***	0.899	0.070***	0.820	0.370***	0.805
USA (26,329,767; +38)	0.171***	0.963	0.219***	0.964	0.037***	0.913	0.208***	0.962
Japan (2,855,360; +36)	0.218***	0.891	0.384***	0.946	0.046*	0.291	0.236***	0.877
Taiwan (539,165; +24)	0.138***	0.788	0.266***	0.851	0.034	0.486	0.162*	0.742
Singapore (17,525; +1)	0.073**	0.513	0.108*	0.366	0.042	0.223	0.129**	0.477
Seychelles (7,010; −5)	0.072	0.249	0.087	0.139	0.158	0.346	0.126	0.270
Australia (1,487,279; −27)	0.202***	0.944	0.292***	0.947	0.029	0.566	0.256***	0.944
South Africa (2,429,415; −29)	0.130***	0.818	0.265***	0.861	0.024	0.181	0.118***	0.800
Chile (429,012; −30)	0.238***	0.863	0.409***	0.883	0.044	0.387	0.285**	0.682
New Zealand (309,220; −41)	0.215***	0.947	-	-	-	-	-	-

Countries are ordered according to mean latitude. CVD: Cardiovascular disease.

* Seasonal variation statistically significant at 5% level;

** Seasonal variation statistically significant at 1% level;

*** Seasonal variation statistically significant at <1% level.

The estimated peak to nadir difference was higher for CVD than for overall mortality. The highest difference towards seasonal CVD mortality was found in South Africa (where the CVD peak to nadir difference was 2.04 times the overall mortality peak to nadir difference) and the lowest value was found in England and Wales, where the CVD peak to nadir difference was 1.08 times the overall mortality peak to nadir difference. The countries most remote from the Equator did not display the greatest peak to nadir values: the peak to nadir differences for CVD and non-CVD/non-cancer mortality were respectively 0.185 and 0.165 in Finland, and 0.266 and 0.199 in Canada (i.e. the countries that were farthest from the Equator in our analysis), whereas these differences were greater in countries that were closer to the Equator, for instance in Portugal (0.466 and 0.370) and in Japan (0.384 and 0.236).

**Figure 2:**
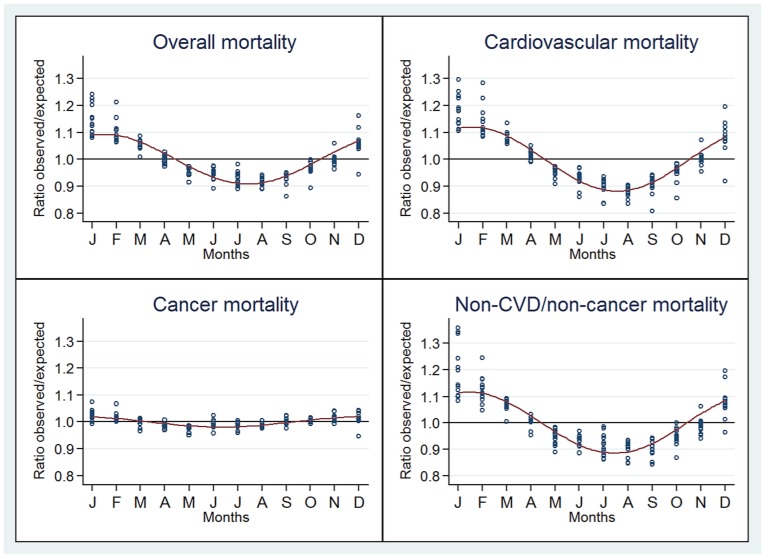
Seasonal variation of the ratio of observed to expected deaths for overall, cardiovascular, cancer and non-CVD/non-cancer. Countries located in the Northern Hemisphere.

**Figure 3:**
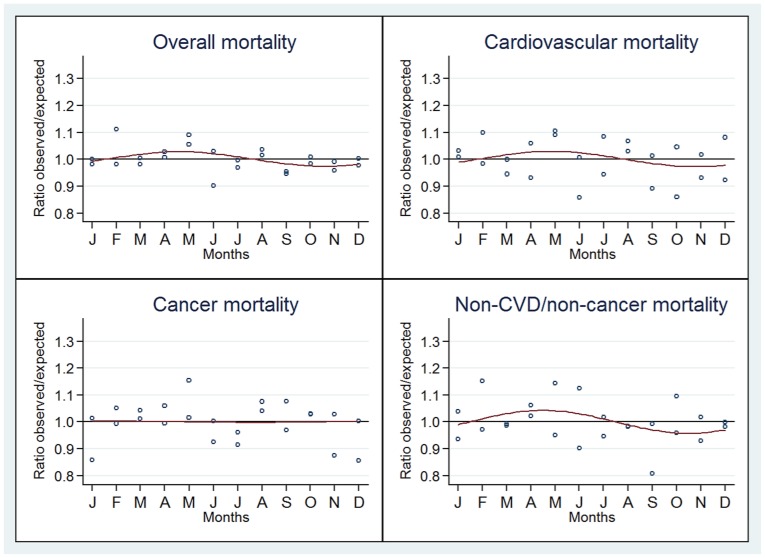
Seasonal variation of the ratio of observed to expected deaths for overall, cardiovascular, cancer and non-CVD/non-cancer. Countries located close to the Equator.

**Figure 4:**
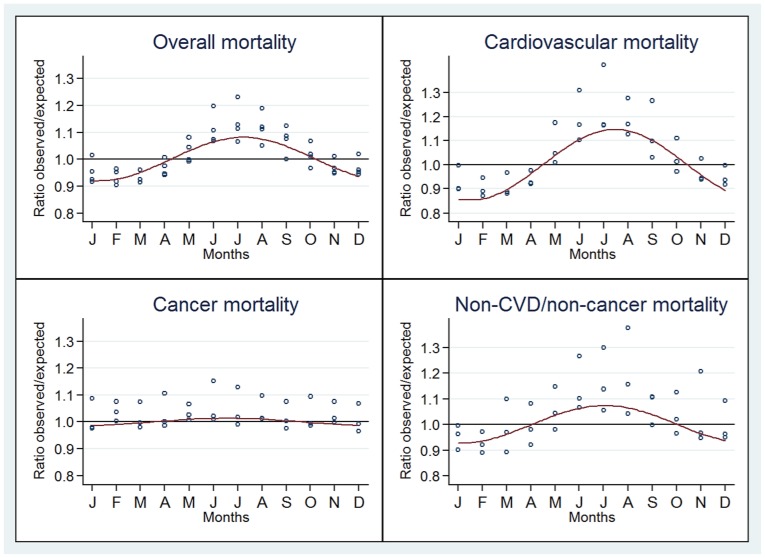
Seasonal variation of the ratio of observed to expected deaths for overall, cardiovascular, cancer and non-CVD/non-cancer. Countries located in the Southern Hemisphere.

The observed/expected mortality ratios for overall and cause-specific deaths according to month and the estimated sinusoidal curve jointly for countries in the same location are represented in **Figure 2** (Northern Hemisphere), **Figure 3** (Equator) and **Figure 4** (Southern Hemisphere). A seasonal pattern was observed for countries in both the Southern and Northern hemispheres and for overall, CVD and non-CVD/non-cancer deaths. Conversely, no or little seasonality was observed in countries close to the Equator for all deaths categories.

## Discussion

Mortality from overall, CVD and non-CVD/non-cancer showed a seasonal pattern, while no consistent seasonality was observed for cancer mortality. Our results also show a gradient of seasonality according to a country' latitude.

### Overall and CVD mortality

Overall and CVD mortality showed a clear seasonal pattern, being higher in winter than in summer. The peak to nadir differences were highest in countries located at mid-distance from the Equator. Our results are in agreement with the few previous studies that investigated the seasonality of overall mortality over a large range of latitudes [2], [12], [18]. In 1999, Douglas and Rawles used the United Nations Demographic Yearbook—which contained some low-quality data, acknowledged by the authors—to get the mortality data of 89 countries between 1976 and 1984. They were the first to describe an inversion of the seasonal pattern of overall mortality between the Northern and Southern hemispheres [18]. Second, Healy investigated the seasonality of overall mortality among 14 European countries. He described the “paradox of excess winter mortality”: the northernmost countries–which have the coldest climates– displayed less seasonality of mortality than some countries at less extreme latitudes in the same hemisphere. He concluded that exposure to cold was not the only factor contributing to seasonality of mortality, and he suggested that socio-economic and housing conditions also played an important role [2]. This hypothesis was further supported by another study from the same author that found large inequalities in housing conditions throughout European countries, which led to the concept of “fuel poverty” [19]. However, these previous studies explored mainly overall mortality and did not address cause-specific mortality. Mechanisms of seasonality of mortality may differ depending on the causes of death. Therefore, we focused in our analysis on seasonality of death according to different broad causes in 19 countries. This allowed us to disentangle components contributing to the seasonality of overall mortality.

The effect of local temperature on seasonality has been shown in geographically limited studies [20], [21]. However, we did not consider this aspect in our analysis because mean temperature at national level would not be very informative in several countries such as Chile or the USA due to the large within-country variations. Moreover, and as mentioned above, the effect of outside temperature on health is modulated at the individual level by various factors such as insulation of houses and quality of clothing people wear to protect themselves against cold, so that people living in countries with very low winter temperatures (e.g. Canada or European Nordic Countries) might actually suffer less from winter cold than people living in warmer countries where houses are less insulated against cold (e.g. Portugal).

Given that mortality from cancer showed virtually no seasonality pattern, the seasonality of overall mortality is driven mostly by seasonality of both CVD and non-CVD/non-cancer mortality. For these conditions, and particularly for CVD, exposure to cold is a plausible explanation for the observed seasonality, given relationship of cold climate with latitude. Several longitudinal studies have demonstrated that a decrease in outdoor temperature was associated with a rise in all cause mortality [2], [22], [23]. However, other latitude-dependent factors, such as dietary habits, sun exposure (vitamin D levels [24]) and human parasitic and infectious agents [25] might also play a role. The magnitude of the seasonal pattern for CVD mortality was highest than that for all cause mortality. The seasonality of CVD mortality might be partly due to the joint seasonality of several known CVD risk factors, as described previously [7]. Similarly, lifestyle factors such as diet [26] and physical activity [27] also tend to differ during summer and winter months. Moreover, exposure to cold increases energy expenditure, peripheral vasoconstriction and cardiac afterload, thus potentially triggering myocardial ischemia [28] and stroke [29]. Finally, winter prone influenza infection might also be a trigger for CVD deaths by exacerbating CVD conditions or due to secondary complications [10], [11]. This is likely to be the case of concentration of air pollutants [30].

The seasonality of non-CVD/non-cancer mortality can relate to the facts that chronic obstructive pulmonary disease and pneumonia are frequent diseases in this category and that these disease are exacerbated by influenza, other influenza-like infections and concentrations of air pollutants, which are all more frequent in winter [30]. A few other diseases in the non-CVD/non-cancer category also present a seasonal pattern, e.g. depression, suicide, and oesophageal variceal bleeding [3], [31].

### Cancer mortality

Deaths from cancer showed little or no seasonal variation in most countries. This result was concordant with a previous study [12]. Factors modulating cancer mortality are different from those modulating CVD. In particular, short time intervals between exposure to risk factors and outcomes is usual for CVD (e.g. instant exposure to cold temperature for selected cardiovascular events) while unusual for cancer, bluring seasonality patterns in the latter. Furthermore, no association was found between amplitude of cancer mortality and latitude, again suggesting that, contrary to CVD mortality, environmental factors such as temperature do not influence cancer mortality.

### Study strengths and limitations

In some countries it was not possible to differentiate deaths from external causes, infectious or respiratory diseases. We decided to create a single category, non-CVD/non-cancer deaths to allow comparisons between countries but this represents a loss of information. A potential limitation relates to the fact that some deaths classified as infectious diseases can be attributed to cardiovascular diseases. However, in a previous study, we showed that hospitalizations for cardiovascular disease also occurred less frequently in summer months [6] and cardiovascular risk factors also showed seasonal variation with increased levels in winter [7]. Furthermore, it seems unlikely that this potential misclassification would impact seasonality equally in all countries. Hence, it is unlikely that the seasonality observed for cardiovascular disease is solely explained by misclassification of deaths from infectious diseases. Second, the availability of mortality data varied between the countries, and it was not possible to collect data for the same period of time for all countries. Data of several years was pooled for a maximum period of ten years for some countries (i.e. Singapore and Seychelles) where the numbers of deaths per month were small. Further, this study was not aimed at assessing secular trends in seasonality of mortality, and restricting the analysis to 5 years periods led to similar results regarding amplitude. A major limitation is related to the fact that countries near the equator are underrepresented in this study. Of note, vital registration in many of these countries is not comprehensive and coverage of deaths less than optimal. The low numbers of deaths from the two small countries close the Equator (Singapore and Seychelles) reduced the power of the seasonality analysis, but they represent the only reliable data we could find in countries close the Equator and such data are essential for a proper analysis according to latitude. Although national mortality data used here were collected according to procedures that may differ between countries, we do not expect differences in the reporting of broad causes of deaths within and between countries, or throughout different months, because all countries use the International Classification of Diseases (ICD); the possible use of different versions of ICD is unlikely to affect the classification of the broad categories of disease considered. However, given their strategic geographic locations, we believe that the included countries are nevertheless a representative sample of the World's countries, and therefore they allow assessing correctly the global seasonality of mortality by major cause of the death.

Finally, mortality data seemed to be, overall, of good quality with few missing data and collection methods well described: This strengthens our observations and conclusions.

### Conclusion

We found a seasonal pattern for mortality from overall, CVD and non-CVD/non-cancer causes and this pattern seemed to vary according to latitude. Conversely, cancer mortality showed weak or no seasonality in all countries studied. Further studies should investigate the determinants of seasonality of mortality by cause of the death.

## Supporting Information

Figure S1 **Distribution of the proportion of deaths from all causes (y-axis) per month (from January to December, x-axis) by country.** The numbers close to the country name refer to the mean latitude of a country. Countries from the Northern Hemisphere close to the Equator and Southern Hemisphere, respectively, in green, red and blue.
(TIF)

Figure S2 **Distribution of the proportion of cancer deaths (y-axis) per month (from January to December, x-axis) by country.** The numbers close to the country name refer to the mean latitude of acountry. Countries from the Northern Hemisphere close to the Equator and Southern Hemisphere, respectively, in green, red and blue. No data available for France and New Zealand.
(TIF)

Figure S3 **Distribution of the proportion of non-cardiovascular/non-cancer deaths (y-axis) per month (from January to December, x-axis) by country.** The numbers close to the country name refer to the mean latitude of a country. Countries from the Northern Hemisphere close to the Equator and Southern Hemisphere, respectively, in green, red and blue. No data available for France and New Zealand.
(TIF)

Table S1 **Characteristics of the countries included in the analysis of seasonality of mortality.**
(DOCX)

Table S2 **Data sources.**
(DOCX)
