# Supplementary figures and images for: Seasonal Variation of Overall and Cardiovascular Mortality: A Study in 19 Countries from Different Geographic Locations
Source: PLoS One. 2014 Nov 24;9(11):e113500. doi: 10.1371/journal.pone.0113500 (PMC4242652; doi:10.1371/journal.pone.0113500)

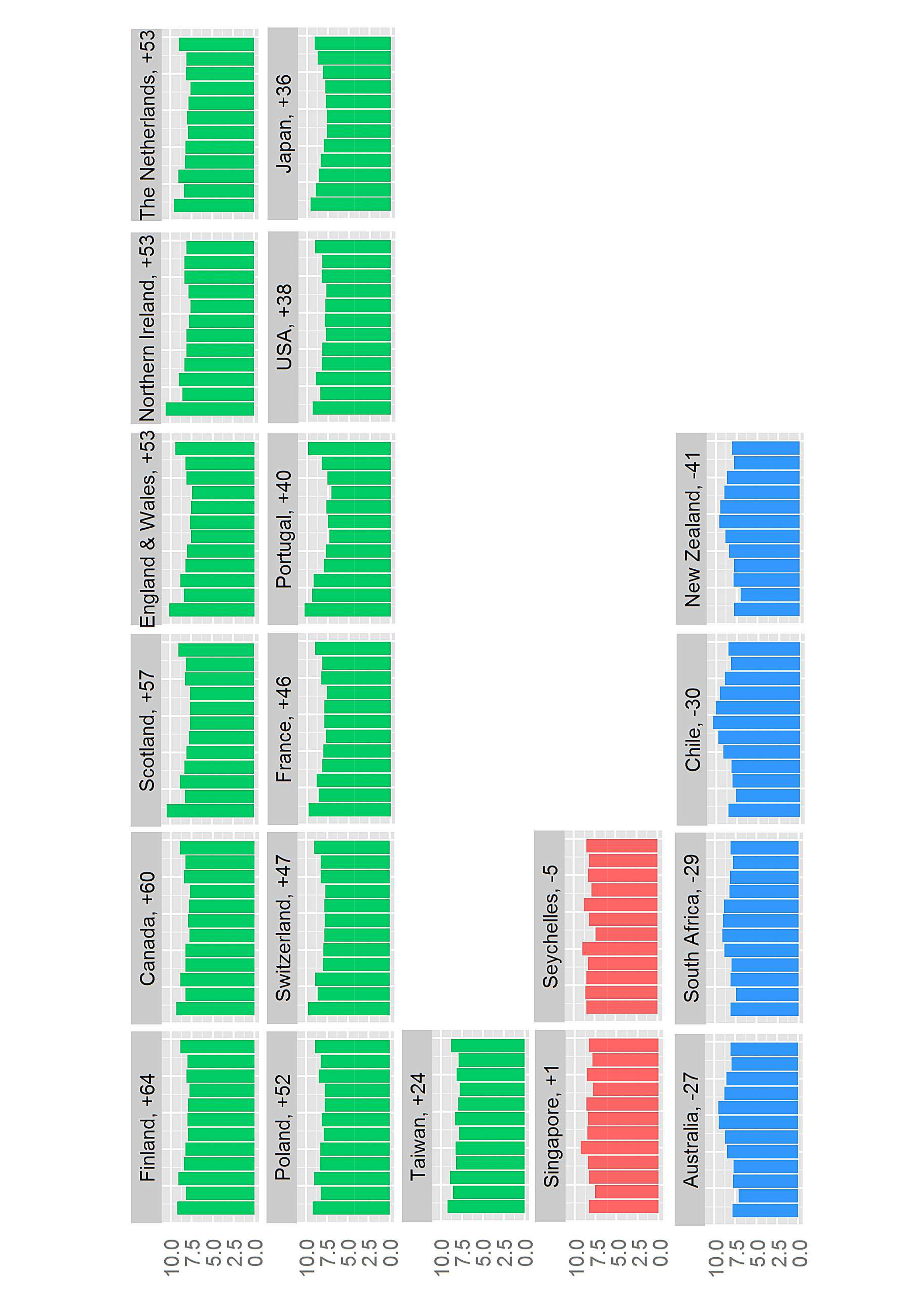

Supplement: Figure S1 — Distribution of the proportion of deaths from all causes (y-axis) per month (from January to December, x-axis) by country. The numbers close to the country name refer to the mean latitude of a country. Countries from the Northern Hemisphere close to the Equator and Southern Hemisphere, respectively, in green, red and blue. (TIF) [file pone.0113500.s001.tif]

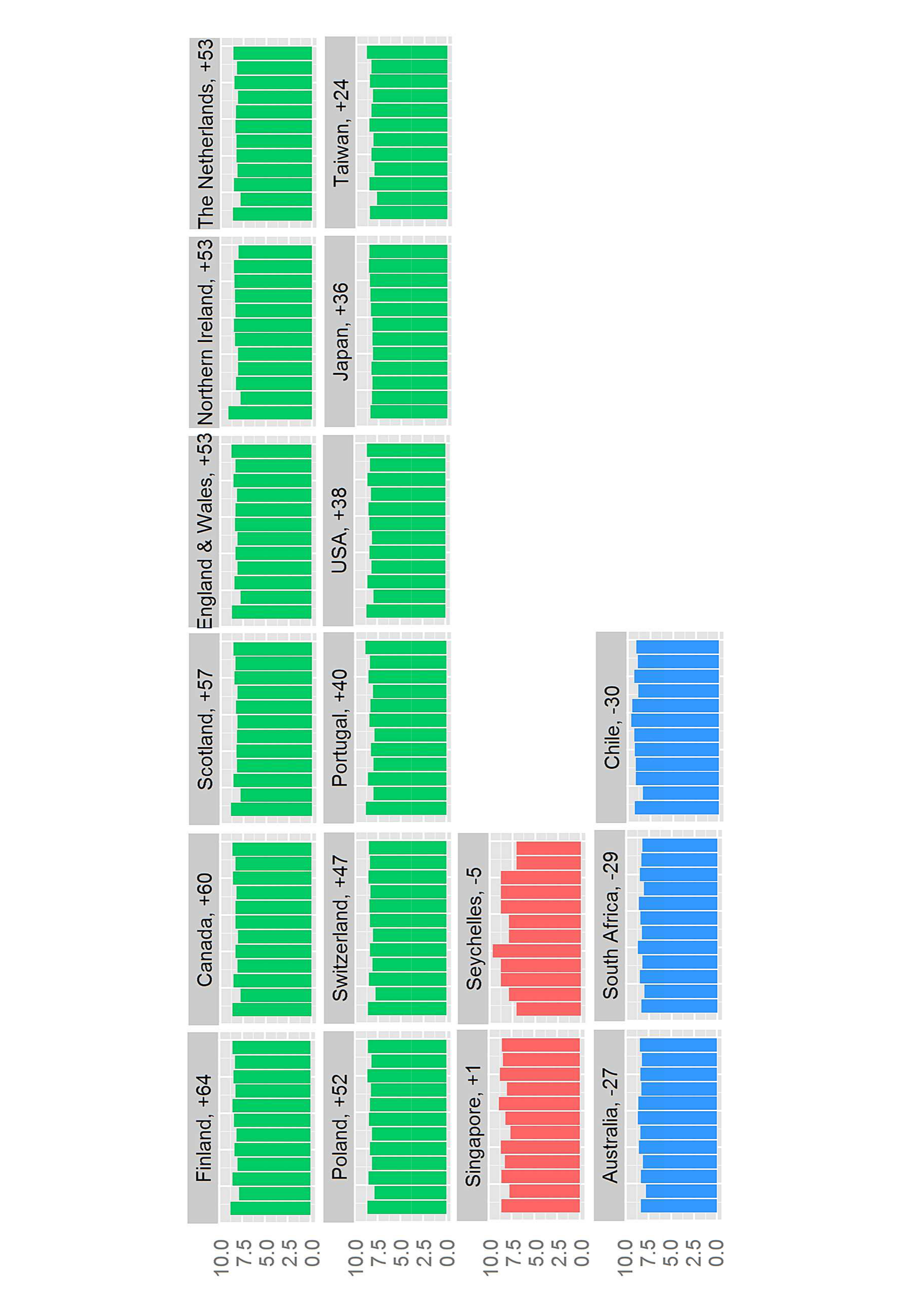

Supplement: Figure S2 — Distribution of the proportion of cancer deaths (y-axis) per month (from January to December, x-axis) by country. The numbers close to the country name refer to the mean latitude of acountry. Countries from the Northern Hemisphere close to the Equator and Southern Hemisphere, respectively, in green, red and blue. No data available for France and New Zealand. (TIF) [file pone.0113500.s002.tif]

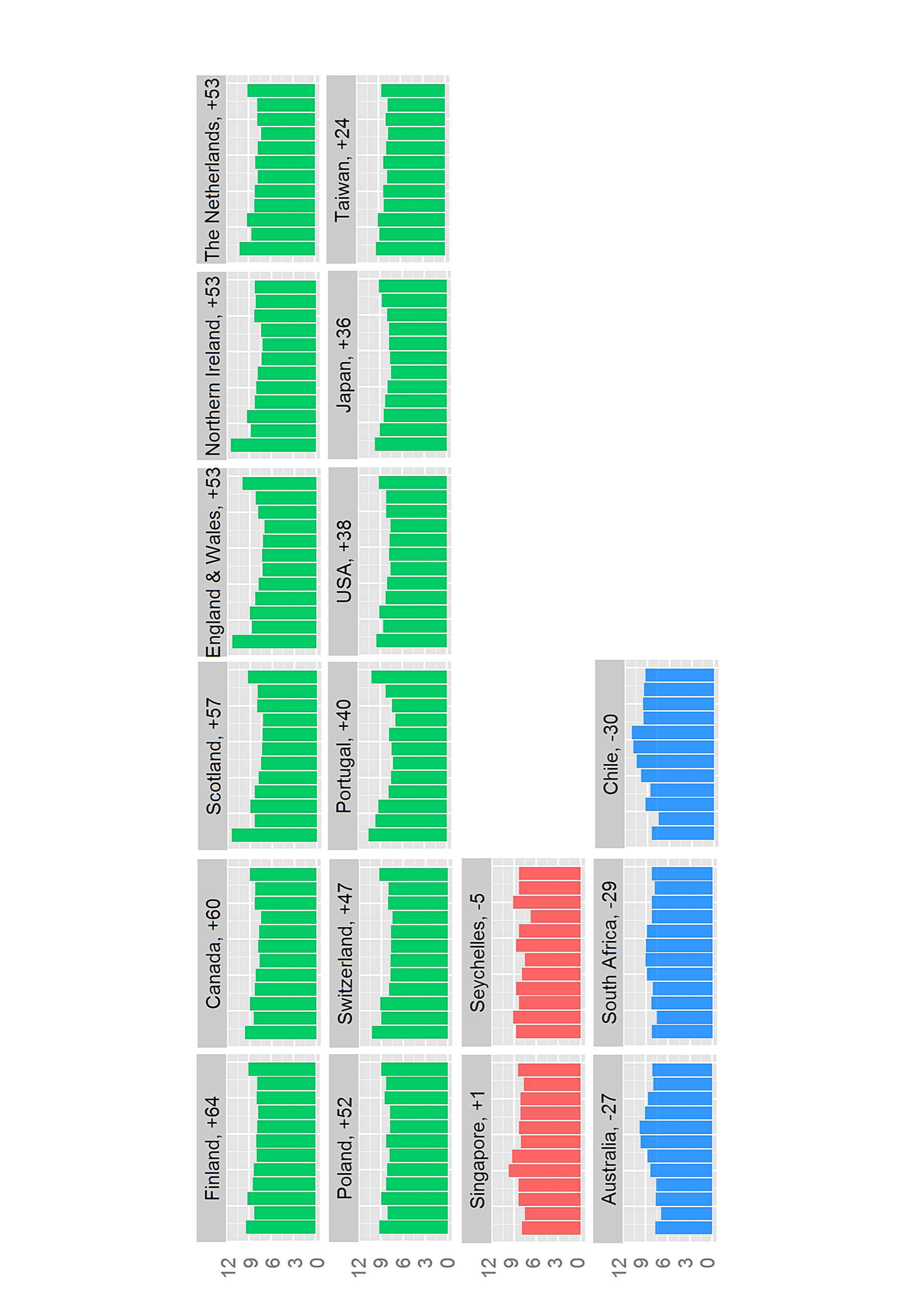

Supplement: Figure S3 — Distribution of the proportion of non-cardiovascular/non-cancer deaths (y-axis) per month (from January to December, x-axis) by country. The numbers close to the country name refer to the mean latitude of a country. Countries from the Northern Hemisphere close to the Equator and Southern Hemisphere, respectively, in green, red and blue. No data available for France and New Zealand. (TIF) [file pone.0113500.s003.tif]
