# Supplementary material for: Seasonal Variation of Overall and Cardiovascular Mortality: A Study in 19 Countries from Different Geographic Locations
Source: PLoS One. 2014 Nov 24;9(11):e113500. doi: 10.1371/journal.pone.0113500 (PMC4242652; doi:10.1371/journal.pone.0113500)
Supplement: Table S1 — Characteristics of the countries included in the analysis of seasonality of mortality. (DOCX) [file pone.0113500.s004.docx]

Table S1. Characteristics of the countries included in the analysis of seasonality of mortality.

| Country (abbreviation) | Average latitude § | Geographic location §§ | Years collected | Total number of deaths |
| --- | --- | --- | --- | --- |
| Australia (AU) | -27 | S | 2000-2010 | 1,487,279 |
| Canada (CA) | 60 | N | 2000-2009 | 2,284,419 |
| Chile (CL) | -30 | S | 2006-2010 | 429,012 |
| England & Wales (GB-EW) | 53 | N | 2000-2010 | 5,652,495 |
| Finland (FI) | 64 | N | 2000-2009 | 487,942 |
| France (FR) | 46 | N | 2000-2010 | 5,947,305 |
| Japan (JP) | 36 | N | 2009-2010 | 2,855,360 |
| New Zealand (NZ) | -41 | S | 2000-2010 | 309,220 |
| Northern Ireland (GB-I) | 53 | N | 2000-2010 | 160,000 |
| Poland (PL) | 52 | N | 2005-2010 | 2,258,014 |
| Portugal (PT) | 40 | N | 2003-2009 | 735,340 |
| Republic of Seychelles (SC) | -5 | E | 2000-2010 | 7,010 |
| Scotland (GB-S) | 57 | N | 2000-2010 | 618,292 |
| Singapore (SG) | 1 | E | 2010 | 17,525 |
| South Africa (ZA) | -29 | S | 2006-2009 | 2,429,415 |
| Switzerland (CH) | 47 | N | 2000-2007 | 487,635 |
| Taiwan (Republic of China) (TW) | 24 | N | 2003-2006 | 539,165 |
| The Netherlands (NL) | 53 | N | 2000-2010 | 1,511,810 |
| United States of America (US) | 38 | N | 2000-2010 | 26,329,767 |

§, according to [[16](#_ENREF_16)]; §§: N, Northern hemisphere; E, Equator; S, Southern hemisphere.
