# Supplementary material for: Seasonal Variation of Overall and Cardiovascular Mortality: A Study in 19 Countries from Different Geographic Locations
Source: PLoS One. 2014 Nov 24;9(11):e113500. doi: 10.1371/journal.pone.0113500 (PMC4242652; doi:10.1371/journal.pone.0113500)
Supplement: Table S2 — Data sources. (DOCX) [file pone.0113500.s005.docx]

Table S2. Data sources.

| Country (abbreviation) | Reference / web site |
| --- | --- |
| Australia (AU) | Australian Institute of Health and Welfare (www.aihw.gov.au/) |
| Canada (CA) | Statistics Canada (www.statcan.gc.ca) |
| Chile (CL) | National Statistics Institute of Chile (www.ine.cl/canales/corporativo/index_corporativo_eng.php?lang=eng) |
| England & Wales (GB-EW) | England and Wales Office for National Statistics (www.ons.gov.uk/ons/index.html) |
| Finland (FI) | Statistics Finland (www.stat.fi/index_en.html) |
| France (FR) | French National Institute of Statistics and Economics (www.insee.fr/en/) |
| Japan (JP) | Statistics and Information Department, Ministry of Health, Labour and Welfare, *Vital Statistics of Japan* |
| New Zealand (NZ) | Statistics New Zealand (www.stats.govt.nz/) |
| Northern Ireland (GB-I) | Census Office for Northern Ireland (www.nisra.gov.uk/Census.html) |
| Poland (PL) | National Institute of Public Health – National Institute of Hygiene  (Prof. Bogdan Wojtyniak, personal communication) |
| Portugal (PT) | Statistics Portugal (www.ine.pt/xportal/xmain?xpid=INE&xpgid=ine_main) |
| Republic of Seychelles (SC) | Ministry of Health, Republic of Seychelles  (Prof. Pascal Bovet, personal communication) [[31](#_ENREF_31)] |
| Scotland (GB-S) | National Records of Scotland (www.nrscotland.gov.uk/) |
| Singapore (SG) | Singapore Statistical Information Service, Department of Statistics (www.singstat.gov.sg/) |
| South Africa (ZA) | Statistics South Africa (www.statssa.gov.za/) |
| Switzerland (CH) | Swiss Federal Office of Statistics (www.bfs.admin.ch/bfs/portal/en/index.html) |
| Taiwan (Republic of China) (TW) | Department of Public Health, Department of Environmental and Occupational Medicine, National Taiwan University  (Prof. Pau-Chung Chen, personal communication) |
| The Netherlands (NL) | Statistics Netherlands (www.cbs.nl/en-GB/menu/home/default.htm) |
| United States of America (US) | United States National Center for Health Statistics (www.cdc.gov/nchs/) |
